# Effects of Negative Attitudes towards Vaccination in General and Trust in Government on Uptake of a Booster Dose of COVID-19 Vaccine and the Moderating Role of Psychological Reactance: An Observational Prospective Cohort Study in Hong Kong

**DOI:** 10.3390/vaccines11020393

**Published:** 2023-02-08

**Authors:** Xinchen Ye, Ho-Hin Lee, Kam-Hei Hui, Meiqi Xin, Phoenix K. H. Mo

**Affiliations:** 1 Centre for Health Behaviours Research, JC School of Public Health and Primary Care, The Chinese University of Hong Kong, Hong Kong, China; 2 Department of Psychology, The Chinese University of Hong Kong, Hong Kong, China; 3 Department of Rehabilitation Sciences, The Hong Kong Polytechnic University, Hong Kong, China

**Keywords:** uptake, a booster dose of COVID-19 vaccine, negative attitudes towards vaccination in general, trust in government, psychological reactance

## Abstract

Uptake of a booster dose of COVID-19 vaccine is effective in preventing infection and severe consequences caused by COVID-19. The present study examined the effects of negative attitudes towards vaccination in general and trust in government on uptake of a COVID-19 booster dose, as well as the moderating role of psychological reactance to pro-vaccination messages in Hong Kong. An observational prospective cohort study using online survey was conducted among 264 adults. Findings showed that, after adjustment for significant background characteristics, negative attitudes towards vaccination in general negatively predicted uptake of a booster dose, and trust in government positively predicted uptake of a booster dose. In addition, the association between negative attitudes towards vaccination in general and uptake of a booster dose was weaker among those who reported a higher level of psychological reactance. The present study highlighted the importance of improving attitudes towards vaccination in general especially among those who are not experiencing psychological reactance, and building trust in government. This study also suggested that interventions aimed at improving attitudes towards vaccination in general should seek to avoid psychological reactance, and special attention should be given to people who are experiencing psychological reactance to pro-vaccination messages.

## 1. Introduction

The COVID-19 pandemic is posing a serious threat around the world. It was reported that 6.9 million people died worldwide from COVID-19 as of May 31, 2022. The estimated number of dead was around 2.5 times larger than the reported number [1]. Although social distancing is always regarded as one of the best ways to reduce transmission rates [2], vaccination still plays an critical role in fighting COVID-19. It is believed that herd immunity cannot be achieved until a sufficiently large proportion of individuals establish acquired immunity through natural infection or immunization with a vaccine [3]. Vaccination is effective in not only preventing COVID-19 infection [4], but also preventing severe consequences caused by natural infection [5]. It has been seen as the potential safest way to end the COVID-19 pandemic [6].

However, how long it will take before COVID-19 herd immunity can be achieved is remains questionable due to at least two reasons. Firstly, there is some evidence that immunity induced by COVID-19 vaccine waned. A 6-month longitudinal prospective study showed that humoral response declined substantially six months after receipt of the second dose of COVID-19 vaccine [7]. Secondly, COVID-19 variants emerge rapidly but the efficacy of vaccination against newly emerging variants seems uncertain. A meta-analysis showed that the efficacy of three major types of COVID-19 vaccines, including mRNA-based vaccine, viral vector vaccine, and inactivated vaccine, decreased when faced with the B.1.617.2 (delta) variant [8].

Considering the waning immunity and the uncertain efficacy of vaccines against newly rapid emerging variants, uptake of a booster dose is of great importance. It has been documented that uptake of a booster dose of COVID-19 vaccine not only ensures persistent immunity, but also contributes to fighting against newly emerging variants [9]. Real-world evidence showed that compared to close contacts who received two doses, those who received a booster dose were less likely to be infected [10]. In addition, it has also been found that uptake of a booster dose contributes to preventing severe consequences. A multicenter observational cohort study showed that uptake of a booster dose offered additional protection beyond full vaccination in preventing death [11]. The benefits illustrated above have prompted many countries and regions to offer a booster dose to their citizens. For example, the UK Government initiated a booster dose programme targeting individuals over 50 and those in a clinical risk group since September 2021 [12]. Hong Kong started to offer a booster dose to citizens for free since 11 November 2021 [13].

Although a booster dose was offered even for free in some countries and regions, insufficient uptake is still a huge challenge for policy makers around the world. A cross-sectional survey of 135,821 US adults aged 18 years or older between 1 December 2021 and 10 January 2022 showed that less than half of fully-vaccinated individuals received a booster dose [14]. In Hong Kong, 54% of people aged between 40 and 59 did not receive a booster dose as of 17 March 2022. The number was even higher among older adults [15]. Thus, understanding predictors of uptake of a booster dose and potential moderators becomes a necessity for countries and regions to introduce policies effective in encouraging uptake of a booster dose.

### 1.1. Attitudes towards Vaccination in General

Attitudes towards performing specific behaviors have been seen by many social psychologists as having critical impact on people’s behaviors. For example, the theory of planned behavior (TPB) included attitudes as a powerful predictor for behavioral intention [16]. Till now, there have been a lot of studies investigating the effect of attitudes towards COVID-19 vaccination on intention to receive a booster dose. For example, An anonymous, cross-sectional, population-based online survey in China found that confidence in the safety and effectiveness of COVID-19 vaccination significantly predicted willingness to receive a booster dose [17]. A cross-sectional study among 2059 healthcare workers in Saudi Arabia showed that perceived benefit brought by a booster dose was positively associated with intention to receive a booster dose [18]. Although improving attitudes towards COVID-19 vaccination seems to be an effective way to increase uptake of a booster dose, it may not be the most cost-effective way as it only targets COVID-19 vaccination. It has been found that attitudes towards vaccination in general can significantly predict public’s reactions to H1N1 vaccine [19]. A study also showed that parents who held more positive attitudes towards vaccination in general were more likely to immunize their daughters against HPV [20]. If attitudes towards vaccination in general can also predict uptake of a booster dose of COVID-19 vaccine, improving attitudes towards vaccination in general may bring more benefit compared to improving attitudes towards COVID-19 vaccination. However, few studies examined how attitudes towards vaccination in general affects uptake of a COVID-19 vaccine booster dose. From our understanding, only a cross-sectional study among adult Americans showed that people who reported a lower level of hesitancy to receive a booster dose were more likely to hold positive attitudes towards vaccination in general [21]. Thus, the effect of attitudes towards vaccination in general on uptake of a booster dose is still worthy of being studied.

### 1.2. Trust in Government

Governments often play a critical role in fighting against pandemic because they are responsible for providing official recommendations. Nevertheless, citizens’ refusal to comply maybe a huge challenge [22]. A lower level of trust in government can lead to reduced support for government action [23]. Thus, it is plausible to infer that individuals who do not trust the local government may refuse to receive a booster dose of COVID-19 vaccine as encouraged by the local government. Till now, the associations between trust in government and willingness to receive a booster dose have been widely examined. These studies showed that a higher level of trust in government predicted a higher level of intention to receive a booster dose [24,25]. However, to our knowledge, there is a dearth of studies investigating the effect of trust in government on actual uptake of a booster dose.

### 1.3. A Potential Moderator-Psychological Reactance to Pro-Vaccination Messages

In addition to testing the effects of attitudes towards vaccination in general and trust in government on receipt of a booster dose, identifying potential moderator is also important, which may help policy makers to design special intervention programmes. Till now, very little is known about the potential factors that may moderate the effects of attitudes towards vaccination in general and trust in government on uptake of a booster dose.

The theory of psychological reactance may contribute to this research gap. According to the theory of psychological reactance, individuals believe that they have freedoms to decide which behaviors they want to perform, and they will intend to regain their freedoms if they feel that their freedoms are threatened [26]. For example, being persuaded, instructed or being forced to do something are all seen as threats to individuals’ freedoms to act [27]. When freedoms are threatened, individuals may act to restore their freedoms. One type of restoration is resisting an advocated behavior, e.g., refusing to receive a flu vaccination after exposure to pro-flu vaccination messages [28]. A review of the literature on COVID-19 vaccination hesitancy warned that resistance to mandates may happen even among originally receptive groups [29]. This implies that pro-vaccination messages may trigger psychological reactance not only among individuals who hold negative attitudes towards vaccination in general or do not trust the local government, but also among those who hold positive attitudes or trust the local government. In this case, if individuals are experiencing psychological reactance to pro-vaccination messages, they may be more likely to refuse a booster dose regardless of their attitudes towards vaccination or level of trust the local government. Thus, it is plausible to infer that the effects of attitudes towards vaccination in general and trust in government on uptake of a booster dose maybe weaker among those who report a higher level of psychological reactance to pro-vaccination messages.

The present study investigated the effects of attitudes towards vaccination in general and trust in government on uptake of a booster dose of COVID-19 vaccine, and whether effects of attitudes towards vaccination in general and trust in government vary across people who report varying levels of psychological reactance. We hypothesized that people who hold more negative attitudes towards vaccination in general are less likely to receive a booster dose; while people who have a higher level of trust in government are more likely to receive a booster dose; and psychological reactance to pro-vaccination messages can play a moderating role.

## 2. Methods

### 2.1. Participants and Procedure

The present study was an observational prospective cohort study conducted in Hong Kong. The inclusion criteria of participants included: (1) Hong Kong residents; (2) aged 18 years or above; (3) have been exposed to messages related to COVID-19 vaccination on any social media platforms (e.g., Twitter, Facebook, etc.) in the past month. This study included the baseline survey and the follow-up survey. Both the baseline survey and the follow-up survey were web-based and self-administered.

The baseline survey was conducted from June to August 2021 when the government had not introduced a booster dose. Eligible participants were invited to participate in the baseline survey via social networking applications (e.g., WhatsApp, WeChat, etc.). Participants who were interested in this study were given a hyperlink or a QR code used to log on to Google Forms including the informed consent and survey questions. Participants could answer survey questions only if they provided their informed consent. The baseline survey took around 20 min to complete. Participants would receive an HKD20 (about USD2.56) e-coupon upon completion of the baseline survey through WhatsApp or email as a token of appreciation for the time they spent.

The follow-up survey was conducted in April 2022 when uptake of a booster dose was introduced though not a prerequisite to enter public spaces. Participants who completed the baseline survey were invited to provide informed consent and answer the follow-up questions via social networking applications through which they received e-coupons last year. An HKD50 (about USD6.42) e-coupon was delivered after completion of the follow-up survey.

Ethical approval was obtained from the ethics committee of the Chinese University of Hong Kong. A total of 411 participants completed the baseline survey, and 264 (64.2%) of them completed the follow-up survey.

### 2.2. Measures

#### 2.2.1. Background Variables

Socio-demographic and background characteristics including age, gender, and education level, were collected at the baseline survey.

#### 2.2.2. Uptake of a Booster Dose of COVID-19 Vaccine

There was one item collecting information on whether the participants had received a booster dose of COVID-19 vaccine at the follow-up survey (yes/no). This is the dependent variable of this study.

#### 2.2.3. Negative Attitudes towards Vaccination in General

Negative attitudes towards vaccination in general were measured using the 12-item Vaccination Attitudes Examination (VAX) Scale at the base line survey (1 = strongly disagree to 5 = strongly agree), which included the following four subscales: mistrust of vaccine benefit, worries over unforeseen future effects, concerns about commercial profiteering, and preference for natural immunity [30]. Sample items included the following: (a) “Vaccines make a lot of money for pharmaceutical companies, but do not do much for regular people”, and (b) “Authorities promote vaccination for financial gain, not for people’s health”. A higher score means more negative attitudes towards vaccination in general. The Cronbach’s α of negative attitudes towards vaccination in general in the present study was 0.81.

#### 2.2.4. Trust in Government

Trust towards the governmental vaccination policy was measured by three items at the baseline survey (1 = strongly disagree to 5 = strongly agree), which were adapted from a validated scale measuring citizen trust in government organizations. All the subscales of the original scale have been used. We selected one item respectively from each of the following subscales: benevolence, competence, and integrity [31]. Sample items included: (a) “The Hong Kong government carries out its duty very well”, and (b) “The Hong Kong government acts in the interest of citizens”. A higher score means a higher level of trust towards the governmental vaccination policy. The Cronbach’s α of trust towards the governmental vaccination policy in the present study was 0.92.

In addition, one item assessed trust towards governmental measures for controlling COVID-19 in general (1 = very distrustful to 5 = very trustworthy).

#### 2.2.5. Psychological Reactance to Pro-Vaccination Messages

Following some other well designed studies [32,33], psychological reactance to pro-vaccination messages on social media, a form of state reactance, were measured by six items at the baseline survey (1 = strongly disagree to 5 = strongly agree), which were adapted from a four-item subscale measuring resisting influence from others and a two-item subscale measuring reactance to advice and recommendations of the 14-item Hong’s Psychological Reactance Scale [34]. Sample items included: (a) “I will try not to let social media influence my willingness to get vaccinated”, and (b) “The suggestions and appeals on social media about vaccinations will make me even more reluctant to vaccinate”. A higher score means a higher level of psychological reactance triggered by pro-vaccination messages. The Cronbach’s α of psychological reactance in the present study was 0.76.

### 2.3. Statistical Analysis

Firstly, descriptive statistics were used to present the background characteristics and prevalence of uptake of a booster dose among participants who completed both the baseline survey and the follow-up survey. Chi-square tests were used to test whether there were differences in the background characteristics between participants who completed both the baseline survey and the follow-up survey and those who only completed the baseline survey. Then, univariate logistic regression was conducted to examine the associations between background variables and uptake of a booster dose, as well as the associations between variables of interest and uptake of a booster dose. We also performed multivariate logistic regression analysis to test the associations between each of variables of interest and uptake of a booster dose adjusted for all the significant background variables. Last, hierarchical regression adjusted for all the significant background variables was used to testing the moderating role of psychological reactance. The main effects of negative attitudes towards vaccination in general and trust in government were first examined. Then the two-way interaction terms between each of the independent variables and psychological reactance were calculated and added to main effect models. Centered scores were used to calculate the interaction terms. Crude odds ratios (ORc), adjusted odds ratios (ORa), and their 95% confidence interval (CI) were obtained. Data analysis was performed using STATA version 15.0 with *p* < 0.05 considered as statistically significant.

## 3. Results

### 3.1. Background Characteristics of the Participants and Prevalence of Uptake of a Booster Dose

The descriptive statistics are presented in Table 1. Of the participants who completed both the baseline survey and the follow-up survey (*n* = 264), over half were females (59.9%), aged 20–39 years old (59.1%), and had a university or above education level (80.7%). 50% of them received a booster dose as of the follow-up survey. There was no significant difference in the background characteristics between participants who completed both the baseline survey and the follow-up survey and those who only completed the baseline survey.

### 3.2. Associations between Background Characteristics and Uptake of a Booster Dose

The associations between background characteristics and uptake of a booster dose are presented in Table 2. Results from univariate logistic regression analysis shows that participants who aged 40–49 (reference = 18–19; ORc = 5.47, 95% CI: 1.68–17.81), aged 50 or above (reference = 18–19; ORc = 4.81, 95% CI: 1.84–12.58), and had a university or above education level (ORc = 0.38, 95% CI: 0.20–0.73) were more likely to receive a booster dose. Gender was not significantly associated with uptake of a booster dose.

### 3.3. Associations between Variables of Interest and Uptake of a Booster Dose

As shown in Table 3, adjusted for all the background factors significantly associated with uptake of a booster dose, people who held more negative attitudes towards vaccination in general (ORa = 0.25, 95% CI: 0.15–0.15) were less likely to receive a booster dose. People who reported a higher level of trust towards the governmental vaccination policy (ORa = 1.75, 95% CI: 1.33–2.31), and reported a higher level of trust towards governmental measures for controlling COVID-19 in general (ORa = 1.53, 95% CI: 1.20–1.96) were more likely to receive a booster dose. Psychological reactance was not significantly associated with uptake of a booster dose after controlling for significant background characteristics.

### 3.4. The Moderating Role of Psychological Reactance to Pro-Vaccination Messages

**Figure 1:**
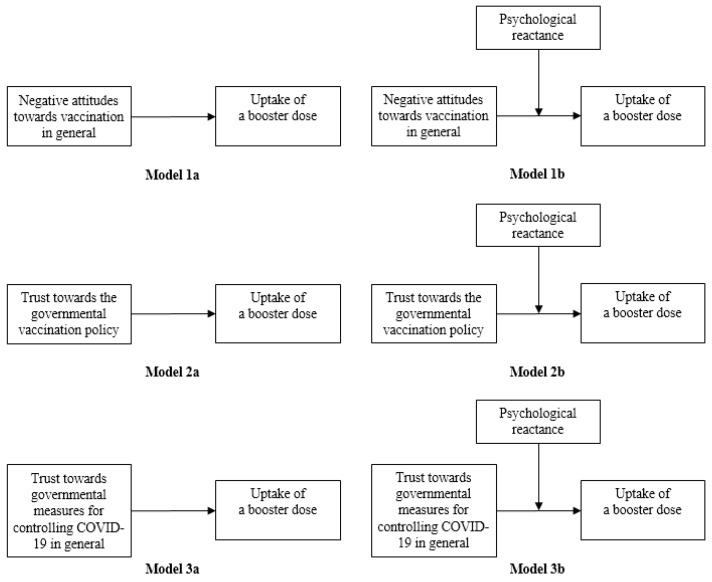
The hypothesized path for each of the hierarchical model.

**Figure 2:**
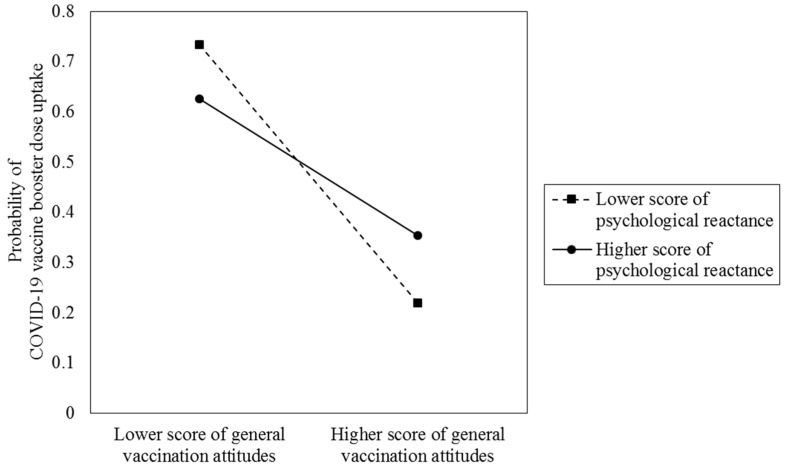
The moderating role of psychological reactance on the effects of negative attitudes towards vaccination in general on uptake of a booster dose (*n* = 264).

Figure 1 shows the hypothesized path for each of the hierarchical model. Results from hierarchical regression analysis are presented in Table 4. Model 1a, Model 2a, and Model 3a estimated the main effects of negative attitudes towards vaccination in general, trust towards the governmental vaccination policy, and trust towards governmental measures for controlling COVID-19 in general on uptake of a booster dose respectively. Model 1b, Model 2b, and Model 3b investigated the moderating role of psychological reactance in such relationships respectively. As results from Model 1a, Model 2a, and Model 3a show, after controlling for psychological reactance and significant background characteristics, there were significant main effects of negative attitudes towards vaccination in general (ORa = 0.24, 95% CI: 0.13–0.44), trust towards the governmental vaccination policy (ORa = 1.71, 95% CI: 1.29–2.28), and trust towards governmental measures for controlling COVID-19 in general (ORa = 1.49, 95% CI: 1.16–1.92) on uptake of a booster dose. After adding each of two-way interaction terms to Model 1b, Model 2b, and Model 3b respectively, we found that psychological reactance significantly moderated the relationship between negative attitudes towards vaccination in general and uptake of a booster dose (ORa = 0.44, 95% CI: 0.21–0.94). The result from single slope analysis is presented in Figure 2. It can be seen that the gaps in uptake of a booster dose between people with more positive attitudes towards vaccination in general and more negative attitudes towards vaccination in general reduced with an increase in psychological reactance.

## 4. Discussion

To our knowledge, this is the first study examining the effects of negative attitudes towards vaccination in general and trust in government on uptake of a COVID-19 booster dose using data from an observational prospective cohort study in Hong Kong. This study found that prevalence of a booster dose uptake was 50% as of the follow-up survey, which was similar to the figure (46.6%) among people aged 20 or above as of 13 April 2022 reported by the Hong Kong government [13]. It has been documented that receiving a booster dose contributes to preventing not only infection, but also negative consequences caused by COVID-19 [9,10,11]. Thus, promoting uptake of a booster dose in Hong Kong is still a necessity.

In the present study, negative attitudes toward vaccination in general at the baseline survey were found to be significantly associated with uptake of a booster dose as of the follow-up survey. More specifically, people who held more negative attitudes at the baseline survey were less likely to report uptake of a booster dose after the Hong Kong government introduced a booster dose. This finding is consistent with studies showed that the more negative attitudes towards vaccination in general, the lower likelihood for people to receive a H1N1 vaccine or immunize their daughters against HPV [19,20]. This finding also provided a possible explanation for the association between history of flu vaccination and COVID-19 booster dose vaccination found in many studies [13,35]. That is, people who hold more positive attitudes towards vaccination in general will be more likely to receive other kinds of vaccines, including flu vaccine, COVID-19 vaccine, and etc. Our study implied that improving attitudes towards vaccination in general may bring more benefit because it can also increase uptake of other kinds of vaccines. Thus, interventions aimed at improving attitudes towards vaccination in general should be designed. In addition, the present study also showed that trust towards both the governmental vaccination policy and governmental measures for controlling COVID-19 in general positively affected uptake of a booster dose. Our findings are in line with the literature that trust towards government were associated with COVID-19 vaccination [36,37]. This finding suggested that building trust in government can play an important role in increasing uptake of a booster dose of COVID-19 vaccine.

Very little research has been conducted to examine whether psychological reactance triggered by pro-vaccination messages can moderate the effects of negative attitudes towards vaccination in general and trust in government on uptake of a booster dose. Our results showed that the relationship between negative attitudes towards vaccination in general and uptake of a booster dose was significantly weaker among people who reported a higher level of psychological reactance. In other words, the gaps in uptake of a booster dose caused by different attitudes towards vaccination in general will reduce if people are experiencing psychological reactance triggered by pro-vaccination messages. The possible explanation may be that if psychological reactance to pro-vaccination messages has been triggered, people will intend to refuse a booster dose no matter what attitudes towards vaccination in general they hold, which is in line with the literature that resistance to mandates may happen even if individuals are receptive at the beginning [29]. Furthermore, if psychological reactance to pro-vaccination messages occurs, people who hold positive attitudes towards vaccination in general may be more likely to be affected compared to people who hold negative attitudes towards vaccination in general, which is plausible because people who hold negative attitudes towards vaccination in general will intend to refuse a booster dose no matter whether psychological reactance to pro-vaccination messages occurs or not, while people who hold positive attitudes towards vaccination in general will intend to receive a booster dose when psychological reactance to pro-vaccination messages is low, and refuse a booster dose when psychological reactance to pro-vaccination messages is high. This finding implied that if psychological reactance to pro-vaccination has been triggered, improving attitudes towards vaccination in general will be less effective in increasing uptake of a booster dose. Thus, it was suggested that interventions aiming at improving attitudes towards vaccination in general should seek to avoid psychological reactance, and special attention should be given to people who are experiencing psychological reactance to pro-vaccination messages.

Findings obtained from the present study have some important implications. Firstly, our findings suggest that improving people’s attitudes towards vaccination in general as well as building people’s trust in government may be effective in increasing uptake of a COVID-19 vaccine booster dose. Given that the four dimensions of negative attitudes towards vaccination in general include mistrust of vaccine benefit, worries over unforeseen future effects, concerns about commercial profiteering, and preference for natural immunity [30], combining a humor correction strategy aiming to attract attention paid to corrective information and a non-humor correction strategy aiming to increase credibility of corrective information to correct misinformation may be helpful [38]. As for building trust in government, making vaccine licensing, manufacture, and prioritization planning transparent may be an effective way [39]. In addition, the present study suggested that improving attitudes may be more effective among people who are not experiencing psychological reactance to pro-vaccination messages, and thus, interventions aimed at improving attitudes towards vaccination in general should seek to avoid psychological reactance. As suggested in existing literature, high-threat language and loss-frame messages should be avoided to minimize psychological reactance [40]. Last but not least, it was suggested that special attention should be given to people who are experiencing psychological reactance to pro-vaccination messages. As for individuals who are experiencing psychological reactance triggered by pro-vaccination messages, a short postscript message reminding the receivers that they have the final choice in their behaviors can be effective in restoring their freedoms and thus reducing psychological reactance they are experiencing [41].

It should be noted that there were some limitations of the present study. First, both the baseline survey and the follow-up survey were self-administered. Thus, there may be some reporting bias, such as recall bias. Second, participants were recruited by non-probabilistic sampling. Therefore, the sample in the present study may not represent the whole adult population in Hong Kong. Third, the present study only studied the effects of negative attitudes towards vaccination in general and trust in government, as well as the moderating role of psychological reactance to pro-vaccination messages. Fourth, the relatively small number of participants in the present study was another limitation. Future studies should test the findings from the present study using a population-representative sample with larger sample size, and examine the effects of other predictors or moderators.

## 5. Conclusions

The present study investigated the effects of negative attitudes towards vaccination in general and trust in government on uptake of a COVID-19 booster dose. It has been observed that both negative attitudes towards vaccination in general and trust in government were associated with uptake of a booster dose, which suggested that improving attitudes towards vaccination in general as well as building people’s trust in government may be effective in increasing uptake of a COVID-19 vaccine booster dose. More importantly, the present study tested the moderating role of psychological reactance to pro-vaccination messages in such relationships. Our findings showed that psychological reactance to pro-vaccination messages significantly weakened the effect of negative attitudes towards vaccination in general on uptake of a booster dose, which suggested that improving attitudes towards vaccination in general may be more effective among people who are not experiencing psychological reactance to pro-vaccination messages. Therefore, it is suggested that interventions aiming at improving attitudes towards vaccination in general should seek to avoid psychological reactance, and special attention should be given to people who are experiencing psychological reactance to pro-vaccination messages.

## Figures and Tables

**Table 1:** Background characteristics of participants by follow-up status (among all participants who completed the baseline survey, *n* = 411) and prevalence of uptake of a booster dose (*n* = 264).

	Follow-Up Completed (*n* = 264)	Lost to Follow-Up (*n* = 147)	All (*n* = 411)	*p*-Value of Chi-Square Test
	*n* (%)	*n* (%)	*n* (%)	
Age group, years				0.122
18–19	30 (11.4%)	7 (4.8%)	37 (9.0%)	
20–39	156 (59.1%)	90 (61.2%)	246 (59.9%)	
40–49	25 (9.5%)	13 (8.8%)	38 (9.3%)	
50 or above	53 (20.1)	37 (25.2%)	90 (21.9%)	
Gender				0.593
Male	106 (40.2%)	63 (42.9%)	169 (41.1%)	
Female	158 (59.9%)	84 (57.1%)	242 (58.9%)	
Educational Level				0.219
Below University	51 (19.3%)	36 (24.5%)	87 (21.2%)	
University or above	213 (80.7%)	111 (75.5%)	324 (78.8%)	
Uptake of a booster dose				N/A
Yes	132 (50.0%)	N/A	N/A	
No	132 (50.0%)	N/A	N/A	

**Table 2:** Associations between background characteristics and COVID-19 vaccine booster dose uptake (*n* = 264).

	Uptake of a Booster Dose	
	ORc (95% CI)	*p*-Value
Age group, years		
18–19	Ref = 1.0	
20–39	1.17 (0.52–2.63)	0.703
40–49	5.47 (1.68–17.81)	0.005
50 or above	4.81 (1.84–12.58)	0.001
Gender		
Male	Ref = 1.0	
Female	1.00 (0.61–1.64)	1.000
Educational Level		
Below University	Ref = 1.0	
University or above	0.38 (0.20–0.73)	0.004

**Table 3:** Factors associated with COVID-19 vaccine booster dose uptake (*n* = 264).

	ORc (95% CI)	*p*-Value	ORa (95% CI)	*p*-Value
Negative attitudes towards vaccination in general	0.21 (0.12–0.37)	<0.001	0.25 (0.15–0.45)	<0.001
Trust towards the governmental vaccination policy	2.02 (1.57–2.59)	<0.001	1.75 (1.33–2.31)	<0.001
Trust towards governmental measures for controlling COVID-19 in general	1.77 (1.41–2.21)	<0.001	1.53 (1.20–1.96)	0.001
Psychological reactance to pro-vaccination messages	0.58 (0.39–0.86)	0.006	0.70 (0.46–1.06)	0.089

Note: Adjusted models were adjusted for significant background characteristics in Table 2.

**Table 4:** Hierarchical regression analysis predicting uptake of a booster dose (*n* = 264).

	ORa (95% CI)	*p*-Value	ORa (95% CI)	*p*-Value
	**Model 1a**		**Model 1b**	
Negative attitudes towards vaccination in general	0.24 (0.13–0.44)	< 0.001	0.22 (0.12–0.42)	< 0.001
Psychological reactance	1.14 (0.70–0.85)	0.596	1.07 (0.66–1.73)	0.787
Interaction term			2.25 (1.06–4.78)	0.035
	**Model 2a**		**Model 2b**	
Trust towards the governmental vaccination policy	1.71 (1.29–2.28)	< 0.001	1.71 (1.28–2.29)	< 0.001
Psychological reactance	0.88 (0.56–1.36)	0.556	0.83 (0.53–1.29)	0.408
Interaction term			0.73 (0.50–1.06)	0.098
	**Model 3a**		**Model 3b**	
Trust towards governmental measures for controlling COVID-19 in general	1.49 (1.16–1.92)	0.002	1.48 (1.15–1.91)	0.002
Psychological reactance	0.82 (0.53–1.25)	0.354	0.78 (0.50–1.20)	0.259
Interaction term			0.80 (0.57–1.14)	0.214

Note: Adjusted models were adjusted for significant background characteristics in Table 2.

## Data Availability

The data was available on reasonable request.
